# Exploring risk and protective factors of intimate partner violence in Korean young adults

**DOI:** 10.1371/journal.pone.0314352

**Published:** 2024-12-02

**Authors:** Soonok An, Jisoo Youn, Qihao Zhan, Soo-Jung Byoun

**Affiliations:** 1 School of Social Work, University of Illinois Urbana-Champaign, Urbana, IL, United States of America; 2 School of Community Health Sciences, Counseling and Counseling Psychology, Oklahoma State University, Stillwater, OK, United States of America; 3 Research fellow, Korea Institute for Health and Social Affairs, Sejong, Republic of Korea; Universidad Pública de Navarra, SPAIN

## Abstract

Intimate partner violence (IPV) is most prevalent in young adults, yet scarce evidence is available regarding South Korean young adults’ experience of IPV and culturally tailored IPV prevention programs. To address this gap, this study aimed to holistically assess IPV victimization and perpetration rates and the related risk and protective factors among Korean young adults. Using online survey data from 600 Korean young adults using simple random sampling, this study found that the lifetime prevalence of both IPV victimization and perpetration was about 30%. Both IPV victimization and perpetration had affected over 20% in the past 12 months. Independent variables in multiple logistic regression models explained 18% and 23% of variances in lifetime IPV victimization and perpetration, respectively. Korean young adults who reported more depressive symptoms were more likely to report IPV victimization. Those who reported more alcohol consumption, traditional attitudes about gender roles, being more tolerant of IPV, and poorer physical health status were also more likely to commit IPV. However, those who had experienced family neglect were less likely to report IPV perpetration. The findings of this study highlighted that childhood adverse experiences minimally explained IPV and that alcohol consumption, mental health, and attitudinal variables should be targets of IPV prevention among Korean young adults.

## Introduction

Intimate partner violence (IPV) is a serious public health issue that affects 11.2% to 27% of South Korean adult men and women [1]. According to the 2021 Korean Violence Against Women Study, IPV constituted about 50% of all forms of violence experienced by South Korean women, generating substantial mental and physical health consequences, including depressive symptoms, anxiety, post-traumatic stress disorder, difficulty trusting others, injury, and physical pain [2]. These consequences can last a lifetime [3]. The need to better prevent IPV among South Korean young adult victims of IPV is clearly urgent.

South Koreans in their 20s face the highest risks of IPV of all age groups, and women have the highest rates [3]; a nationally representative study of women found that among those aged 20–29, rates of physical, sexual, emotional, and controlling abuse ranged from 30% to 51% [2]. These findings aligned with those of studies conducted in the United States [4,5]. However, whereas IPV prevention programs targeting young adults (including college students) are common in Western countries [6–8], very limited information is available to inform contextually appropriate IPV prevention programs for those in South Korea.

Greater attention has addressed IPV among groups that are predominantly older. The 2018 Framework Act on Prevention of Violence Against Women [9] mandated a national survey on IPV. Conducted by the Ministry of Gender Equality of South Korea, the survey focused on married couples [10,11]. By holistically assessing various individual and environmental predictors of IPV among South Korean young adults, this study addressed an urgent need to inform future IPV prevention programs and their implementation.

### Individual risk and protective factors

According to a Korean national study on domestic violence, the prevalence of IPV victimization was comparable among female and male adults in certain categories, such as neglect, coercive control, and emotional abuse. However, economic and physical IPV were generally infrequent but disproportionately affected women [1]. The 18 to 35 age group experienced the highest rates of IPV victimization among South Korean women [3]. A study examining gender-specific IPV experiences among Korean college students revealed no significant gender difference regarding physical and psychological IPV victimization and perpetration [12].

Prior literature has shown that education and a high level of family and personal life satisfaction are individual protective factors against IPV victimization and perpetration among both male and female South Korean adults [13]. A study conducted with South Korean college students revealed that those raised in a two-parent household were less likely to report psychological and physical IPV victimization and perpetration [12]. Having a higher monthly income (about 3,000 U.S. dollars or above) and a white-collar office job also reduced the rate of IPV victimization among Korean women [11,13]. However, neither female nor male college students who grew up in a wealthy family were less likely than others to experience either IPV victimization or perpetration [13].

Regarding individual behavioral risk factors, high rates of risky sexual behaviors and low self-control consistently emerged as determinants of IPV victimization and perpetration among South Korean college students [12,14,15]. In line with the self-control theory, individuals characterized by low self-control and limited self-regulation ability exhibited a higher propensity for subjecting their intimate partners to violent behaviors [16]. Among health behaviors, smoking was not associated with any types of IPV perpetration. However, a study found that alcohol intake was a high-risk factor for IPV perpetration among both male and female Korean adults [13]. A significant association was found between alcohol intake and IPV victimization among Korean males but not females [13].

Research on health risk factors associated with IPV among South Korean young adults has been limited. However, a study found that depressive symptoms were highly associated with psychological and physical IPV perpetration among Korean university students [17]. A review of studies conducted in the United States found that mental health symptoms (e.g., anxiety and depressive symptoms) were risk factors for dating violence victimization and perpetration, especially perpetration [18].

In terms of belief systems or attitudes, a study of South Korean college students found that those with progressive attitudes toward sex roles and a high level of sexual assertiveness were more likely to have a high awareness of dating violence than those without such attitudes [19]. A study of Korean adults aged 19 and up found that women who held positive attitudes toward traditional gender roles and acceptant attitudes of IPV reported higher incidence rates of IPV victimization and that men with those views were more likely to have perpetrated IPV [11]. The same study found that men whose beliefs normalized violence were more likely to engage in violent behaviors toward their parents as well. A study with Korean male university students found that those with acceptant attitudes toward dating violence and toward patriarchal gender roles were more likely to have committed IPV than others [20].

### Environmental risk and protective factors

Adverse childhood experiences (ACEs) have been identified as environmental risk factors strongly related to IPV, and several studies have addressed this relationship among South Korean young adults (e.g., [17]). Among these, studies of college students have found that child maltreatment and witnessing parental IPV towards male and/or female caregivers in childhood increase the risk of IPV perpetration and victimization [1,13,21,22]. One study found that IPV victimization and perpetration rates were three times higher among participants who had witnessed parental IPV during childhood and that female adult IPV victims were twice as likely to have experienced childhood maltreatment than women who had not experienced IPV [11]. Rates of witnessing parental IPV toward male caregivers were significantly lower than rates of witnessing parental IPV toward female caregivers [14]. Moreover, the impact of witnessing parental IPV revealed gender differences toward their adult children. A study conducted in South Korea found that witnessing parental IPV directed at male caregivers had a positive association with IPV perpetration among male college students, while the association was negative for females. This means that for male students, those who observed IPV directed at their fathers were more likely to engage in IPV themselves. However, the effect was the opposite for female students, as witnessing such violence was associated with a lower likelihood of IPV perpetration. The observation of parental IPV towards male caregivers was significantly related to psychological perpetration among male college students and physical perpetration among females [12].

Studies explored mediation and moderation effects on the relationship between IPV and ACEs among South Korean college students. Beliefs that rationalize illegal behaviors mediated the association between experiences of child abuse, witnessing parental IPV, and perpetrating dating violence [21]. On the other side, witnessing parental conflict and guilt-proneness were both independently related to dating violence perpetration [22]. The sense of guilt moderated the effects of witnessing parental conflicts on emotional IPV perpetration [22]. This suggested that a heightened sense of guilt acted as a protective factor, reducing the likelihood that the witnessed parental conflict would translate into emotional aggression in dating relationships. These results underscored the significant role that beliefs and emotional responses play in the intergenerational transmission of violence, particularly within the context of IPV.

No study focusing on the South Korean young adult population has examined the impact of IPV training on IPV victimization and perpetration. However, U.S. studies have consistently found that bystander education and awareness-raising prevention programs are effective in changing attitudes and beliefs toward IPV (e.g., [23]). A study in South Korea reported that awareness of dating violence reduced the risk of female IPV victimization and male IPV perpetration [11]. Dating violence awareness was differed by gender; female Korean college students reported a higher level of dating violence recognition than their male counterparts [19].

### Gaps in the literature

Prior studies on South Korean young adult intimate partner violence have provided adequate evidence of risk and protective factors across different levels. However, most studies have relied on convenience samples from large Korean universities (e.g., [20] or national surveys of married individuals, e.g., [11]). This approach may exclude young adults who are neither married nor enrolled in college, limiting diversity in terms of regions, backgrounds, and socioeconomic status. Furthermore, while prior research on traumatic events and IPV among the South Korean young adult population has predominantly centered on adverse childhood experiences (ACEs) [1,12,21,22], the inclusion of variables related to positive childhood family experiences can provide more comprehensive understanding of both protective and risk factors.

To gain a deeper understanding of how different types of traumatic childhood experiences influence intimate relationships in young adulthood, this study aims to identify lifelong correlates and preventive measures of IPV among South Korean young adults and their environments–findings which future work can translate into interventions and resources that help prevent IPV and mitigate its impact on IPV survivors. Specifically, these findings will provide new information on IPV experience among South Korean young adults that is necessary for developing a culturally tailored IPV prevention program.

### Current study

The person-in-environment perspective [24,25] and cumulative life course adversity models [26,27] served as the conceptual framework for our study examining various individual and environmental risk and protective factors of IPV among South Korean young adults. While individual-level factors largely influence individuals’ thoughts and behaviors, Bronfenbrenner’s ecological systems model advanced a more comprehensive approach to understanding humans’ growth and development within their physical and social environments [28]. Previous studies on IPV have suggested the need to integrate a multilevel approach when exploring the causes of violent behaviors and implementing intervention programs by identifying community- and environmental-level risk factors [24]. In addition, the cumulative life course adversity model demonstrates the traumatic experiences individuals have faced over their lifetimes and assumes that cumulative adversity influences an individual’s lifespan as a whole [27]. Research demonstrates a close connection between multilevel ACEs and exposure to IPV in adulthood (e.g., [29]). The current study also hypothesizes that this connection holds true for Korean young adults in the sample.

This study aims to answer the following research questions with respect to South Koreans aged 19 to 26: 1) What are their lifetime and the past 12-month IPV victimization and perpetration experiences? 2) What are the individual and environmental risk and protective factors affecting those experiences? The study hypothesizes that higher scores on individual factors such as traditional gender role attitude and substance use and lower scores on factors such as knowledge about IPV and health status would be associated with increased risk of IPV victimization and perpetration. The study also hypothesizes that higher scores on environmental factors of childhood adversities such as family neglect, witnessed IPV between caregivers, and experienced abuse and lower scores on family support will be associated with increased risk of IPV victimization and perpetration.

## Methods

### Design and sample

This study used a cross-sectional online survey design with a simple random sampling strategy and obtained survey data from *N* = 600 South Koreans aged 19 to 26 years old in February 2023. The sample size was determined and overestimated by predisposing a .05 effect size at .05 alpha level and .95 power by assuming 20 predictors to run regression models to test this study’s research questions. The sampling frame of this study was a panel (N = 255,929) recruited by an online data collection company in South Korea. The study invited eligible participants based on quota from the panel by eligible participants’ age categories (19–22 and 23–26), binary gender, and 16 regions using the January 2023 national population statistics (N = 4,763,272) published by the Korean Ministry of the Interior and Safety [30]. Our sampling frame consisted of about 5.4% of the population frame. An email invitation was sent to a total of 7,806 target participants, and the survey was closed once 600 complete responses had been received. The response rates were 7.7% of the participants who received the email and 49.1% of those who opened the email. Participants received 5,500 Korean won ($4.41) as an incentive at the completion of the survey. This study was approved by the institutional review board at the author’s institution in January 2023. Each participant reviewed and signed a written, online consent form by clicking a “submit” button before they took the online survey. The online electronic consent form was not documented. Respondents had the option to check “I wish not to respond” for all items related to IPV (CTS-2), childhood adverse experience, and their sexual orientation.

### Measures

#### Lifetime IPV victimization and perpetration

Lifetime IPV victimization and IPV perpetration were measured by the Conflict Tactics Scale (CTS) Short Form [31]. The IPV victimization and IPV perpetration measures consisted of 8 items each that asked about respondents’ lifetime experiences with physical injury (2 items each: e.g., “I had a sprain, bruise, or small cut, or felt pain the next day because of a fight with my partner” or “my partner had a sprain, bruise, or small cut or felt pain the next day because of a fight with me”), physical abuse (2 items each; e.g., “I pushed, shoved, or slapped my partner” or “my partner pushed, shoved, or slapped me”), psychological abuse (2 items each: e.g., “I insulted or swore or shouted or yelled at my partner” or “My partner insulted or swore or shouted or yelled at me), and sexual violence (2 items each; e.g., “I used force to make my partner have sex” or “my partner used force to make me have sex”) in any relationships with a former or current intimate partner. Items were designed as an 8-point scale that indicates the frequency of different types of IPV experience: 0 = this has never happened, 1 = once in the past 12 months, 2 = twice in the past 12 months, 3 = 3–5 times in the past 12 months, 4 = 6–10 times in the past 12 months, 5 = 11–20 times in the past 12 months, 6 = more than 20 times in the past 12 months, 7 = not in the past 12 months, but it did happen before.

Two authors who are bilingual in Korean and English translated the original English version of CTS short form into Korean and thoroughly examined translational accuracy and cultural sensitivity of the Korean CTS short form. Copyright of the use of the Korean CTS short form was obtained from Western Psychological Services. Cronbach’s alpha of IPV victimization and IPV perpetration for the current study were .90 and .91, respectively. Binary versions of the IPV victimization and IPV perpetration variables were created with respect to IPV in participants’ lifetime and in the past 12 months (0 = never experienced IPV or perpetrated it, 1 = ever experienced IPV victimization or perpetrated IPV). A binary variable was also created to measure having experienced IPV victimization and perpetrated it (0 = experienced neither or just one, victimization or perpetration, 1 = have both perpetrated IPV and experienced it).

#### Individual risk and protective factors

**T**he demographic independent variables collected in the survey were age, binary gender, sexual orientation, education level, monthly income, and the number of intimate partners in lifetime. Age, average monthly income in the past 12 months, and the number of intimate partners in lifetime were continuous variables. Gender and sexual orientation were categorized into binary values: 0 = male and 1 = female and 0 = heterosexual and 1 = non-heterosexual, including gay, bisexual, questioning or unsure, and other. Education level was assessed as 0 = high school graduates, 1 = in college, 2 = college graduates or higher.

Two attitude variables included attitudes towards IPV and attitude towards gender roles. This study measured attitudes towards IPV by borrowing items from the IPV Attitude Scales [32]. The scale consisted of 9 items using a 5-point Likert scale (0 = strongly disagree, 1 = somewhat disagree, 2 = neither agree nor disagree, 3 = somewhat agree, 4 = strongly agree). Example statements included “I would not like for my partner to ask me what I did every minute of the day” and “I would never try to keep my partner from doing things with other people.” Scores ranged from 0 to 36, and a higher score indicates greater knowledge about IPV.

Attitude towards gender roles was adopted from a Korean gender role scale used in the 2021 Violence Against Women Survey Study [2]. The scale consists of 10 items using a 4-point Likert scale (1 = strongly disagree, 2 = somewhat disagree, 3 = somewhat agree, 4 = strongly agree). Example items included “making money and doing household chores are work for people of any gender” and “men should be able to be financially responsible for their families” (which was reverse coded). We deleted two items that presented low factor loadings through principal factor analysis, and Cronbach alpha reliability of the final 8 items was .73. Scores range from 13 to 32, and a higher score indicates greater belief in equality in gender roles.

Two substance use variables measuring alcohol consumption and smoking were assessed by two items that were adopted from questions distributed by the U.S. National Institute on Drug Abuse: “In the past 12 months, how often have you used four (4) or more alcohol drinks” and “In the past 12 months, how often have you used tobacco products.” Responses were coded as 0 = “never,” 1 = “once or twice,” 2 = “monthly,” 3 = “weekly”, and 4 = “daily or almost daily.” Score ranges were between 0 and 4, and a higher score indicates that respondents engaged more frequently with alcohol or smoking.

Two health-related variables were assessed in the survey including mental health status and physical health status. Mental health status was evaluated using a 9-item Patient Health Questionnaire (PHQ-9). PHQ-9 measured the frequency of experiencing depressive symptoms over the last two weeks based on 4-point Likert scale (0 = not at all, 1 = several days, 2 = more than half the days, and 3 = nearly every day). Scores range from 1 to 27. The Cronbach alpha reliability of PHQ-9 in this study was .88. Physical health was measured using five items adopted from the Short Form Health Survey using a 5-point Likert scale [33]. Example statements included “I am as healthy as anybody I know” and “my health is excellent” and a higher score indicated better perceived physical health (range: 5–25). Cronbach alpha of this scale was .84 in this study.

#### Environmental risk and protective factors

Environmental risk factors included recent traumatic experience and adverse childhood experiences, including family neglect (8 items, Cronbach alpha = .89), witnessed IPV towards female caregiver (4 items, alpha = .94), witnessed IPV towards male caregiver (4 items, alpha = .92), experienced physical abuse (5 items, alpha = .94), and experienced sexual abuse (4 items, alpha = .89). Recent traumatic experience questions addressed seven experiences in the past 12 months such as divorce/separation, losing a job, and death of a loved one. The score ranges from 0 and 7 and a higher score indicates a higher level of recent traumatic experience. ACE measures asked about cumulative adversities that respondents had experienced before the age of 19 using the Kaiser Scale published by the Centers for Disease Control [34]. Answers to the items were categorized into 5-point responses (0 = never true, 4 = very often true), and higher scores in each variable indicated a higher frequency of experiencing each type of childhood adversities. The instruments’ Cronbach’s alphas were exceptional, ranging from .89 to .94.

Family support before 19 years of age and IPV training experience were assessed as environmental protective factors. Family support was measured using the Kaiser Scale [34]. Family support items (6 items) were asked following the family neglect items; they used a 5-point Likert Scale (0 = never true, 4 = very often true). Family support score ranged from 0 to 24 and a higher score indicated that the respondents experienced a higher level of family support. Cronbach’s alpha of family support was .89. IPV training experience was assessed by asking participants to indicate all types of training experiences focusing on dating, domestic, or intimate partner violence, sexual assault, healthy relationships, toxic relationship, and others. The number of training types were summed, and a higher score meant more types of educational experience related to IPV.

### Data analyses

The study used descriptive statistics to explore participants’ individual and environmental characteristics that are potentially risk or protective factors associated with IPV victimization, IPV perpetration, and experiences both in the past 12 months and lifetime. Hierarchical logistic regression analyses examined the effects of participants’ individual and environmental factors of IPV victimization, IPV perpetration, and experience of both in participants’ lifetime. The first step included individual factors such as sociodemographic characteristics, attitude variables, substance use variables, and health variables. The second step was adding childhood protective and adverse environmental factors, traumatic events in the past 12 months, and IPV training experience. The independent variables in the regression models did not present multicollinearity issues. To conduct logistic regression analyses, *R*^*2*^ statistics were calculated following Nagelkerke’s pseudo *R*^*2*^ formula. All analyses were conducted using *stats*, *Quantpsych*, and *rms* packages in R 4.2.2.

## Results

### Participant characteristics

Sociodemographic characteristics of the respondents are presented in Table 1. Respondents’ mean age was 22.85 years with men comprising 52.0% of respondents and 88.0% of respondents were heterosexual. Average monthly income in the past 12 months was 2,207,300 Korean won (about 1,689.74 U.S. dollars). The income sources that respondents reported (with the option to select more than one) were Employment or related work (64%), Gift from caregiver (63.5%), Scholarship (22.5%), Investment (18%), and Other (1.83%). Average score in the attitudes towards gender roles scale was 31.38, which can be interpreted that overall participants believe in gender equality (range: 18–40). The average score on the attitudes towards IPV scale was 27.49, indicating a generally high level of knowledge about IPV (range: 0–36). The average score on the depressive symptom scale was 5.26, indicating an average person experiencing mild depressive symptoms based on the Patient Health Questionnaire-9 clinical guideline.

**Table 1:** Participant characteristics (N = 600).

	Frequencies N(%) or *M(SD)*	Range
***Individual characteristics***		
Age	*22*.*85(2*.*27)*	19–26
Gender		
Men_0	312(52.0)	
Women_1	288(48.0)	
Sexual orientation		
Heterosexual _0	523(88.0)	
Other_1	71(12.0)	
Education		
High school graduates	79(13.2)	
In college	328(54.7)	
College graduates or more	193(32.2)	
Marital status		
Single	590(98.3)	
Married	10(1.7)	
Average monthly income in the past 12 months	*2*,*207*,*300* *(4*,*857*,*900)*	0–3000
The number of intimate partners in lifetime	*3*.*55(3*.*51)*	1–30
Attitude towards gender roles	*31*.*38(4*.*29)*	18–40
Attitude towards IPV	*27*.*49(6*.*11)*	0–36
Substance use		
Alcohol	*1*.*45(1*.*17)*	
Smoke	.*83(1*.*51)*	
Depressive symptoms	*5*.*26(5*.*04)*	0–27
Physical health	*16*.*90(4*.*06)*	5–25
***Environmental characteristics***		
Family support	*17*.*21(6*.*08)*	0–24
Adverse childhood experiences		
Family neglect	*3*.*92(5*.*41)*	0–28
Witnessed parental IPV towards male caregiver	.*48(1*.*59)*	0–11
Witnessed parental IPV towards female caregiver	.*89(2*.*45)*	0–16
Experienced physical abuse	*2*.*28(4*.*00)*	0–15
Experienced sexual abuse	.*82(2*.*16)*	0–12
training experience related to IPV	*2*.*80(2*.*03)*	0–6
Traumatic events in the past 12 months	*4*.*23(2*.*59)*	2–15

### Rates of intimate partner violence victimization and perpetration

The rates of intimate partner violence (IPV) victimization, perpetration and co-experience (i.e., both victimization and perpetration) by IPV types are presented in Table 2. Over 23% of the study participants reported at least one incident of IPV victimization experience in the past 12 months and almost as many, 21.1% reported IPV perpetration experience in the same period. The participants who experienced both IPV victimization and perpetration was 17.6%. Lifetime IPV victimization, IPV perpetration, and co-experiences of IPV victimization and perpetration rates were 33.9%, 29.7%, and 24.0%, respectively. While lifetime IPV rates were higher than those in the past 12 months, at least 70% of the respondents who reported IPV in their lifetime reported experiencing it in the past 12 months. Psychological abuse victimization was the most common form, followed by physical abuse, sexual abuse, and physical injury, in both the past 12 months and in respondents’ lifetimes, and these results held true across victimization, perpetration, and co-experience of victimization and perpetration in psychological abuse, physical abuse, and injury. About one in four participants had experienced at least one incident of psychological abuse victimization or perpetration in their lifetime. While victimization and perpetration of psychological and physical abuse and physical injury were reported by similar portions of participants, twice as many participants reported sexual abuse victimization as sexual abuse perpetration, both in the past 12-month timelines and in the lifetime.

**Table 2:** Rates of IPV victimization and perpetration (N = 600).

	*In the past 12 months*			*Lifetime*		
	*Yes* *N(%)*	*No* *N(%)*	*Total* *N(%)*	*Yes* *N(%)*	*No* *N(%)*	*Total* *N(%)*
Any IPV						
IPV victimization	**124** **(23.22)**	410 (76.78)	534 (100.00)	**184** **(33.89)**	359 (66.11)	543 (100.00)
IPV perpetration	**113** **(21.08)**	423 (78.92)	536 (100.00)	**162** **(29.72)**	383 (70.28)	545 (100.00)
Co-experience of IPV victimization and perpetration	**93** **(17.58)**	436 (82.42)	529 (100.00)	**130** **(23.99)**	412 (76.01)	542 (100.00)
Physical injury						
Victimization	**48** **(8.63)**	508 (91.37)	556 (100.00)	**65** **(11.67)**	492 (88.33)	557 (100.00)
Perpetration	**40** **(7.23)**	513 (92.77)	553 (100.00)	**55** **(9.95)**	498 (90.05)	553 (100.00)
Co-experience	**36** **(6.56)**	513 (93.44)	549 (100.00)	**44** **(8.00)**	506 (92.00)	550 (100.00)
Physical aggression						
Victimization	**51** **(9.01)**	515 (90.99)	566 (100.00)	**72** **(12.70)**	495 (87.30)	567 (100.00)
Perpetration	**60** **(10.56)**	508 (89.44)	568 (100.00)	**77** **(13.53)**	492 (86.47)	569 (100.00)
Co-experience	**44** **(7.79)**	521 (92.21)	565 (100.00)	**56** **(9.89)**	510 (90.11)	566 (100.00)
Psychological abuse						
Victimization	**96** **(17.55)**	451 (82.45)	547 (100.00)	**135** **(24.59)**	414 (75.41)	549 (100.00)
Perpetration	**104** **(18.98)**	444 (81.02)	548 (100.00)	**135** **(24.46)**	417 (75.54)	552 (100.00)
Co-experience	**80** **(14.81)**	460 (85.19)	540 (100.00)	**100** **(18.42)**	443 (81.58)	543 (100.00)
Sexual abuse						
Victimization	**58** **(10.10)**	516 (89.90)	574 (100.0)	**75** **(13.07)**	499 (86.93)	574 (100.0)
Perpetration	**26** **(4.51)**	550 (95.49)	576 (100.0)	**31** **(5.38)**	545 (94.62)	576 (100.0)
Co-experience	**23** **(4.01)**	550 (95.99)	573 (100.0)	**26** **(4.53)**	547 (95.46)	573 (100.0)

### Individual and environmental risk and protective factors of lifetime IPV victimization

Table 3 presents the results of the two-step hierarchical logistic regression analysis that explored individual and environmental risk and protective factors of lifetime IPV victimization among South Korean young adults. Model 1 included (1) sociodemographic variables such as age, gender, sexual orientation, education level, monthly income, and the number of intimate partners, (2) attitude variables regarding gender roles and IPV, (3) substance use variables such as alcohol and smoking behavior, and (4) health variables such as depressive symptoms and physical health status. The model with individual characteristics accounted for 13.3% of the variance in lifetime IPV victimization (pseudo *R*^*2*^ = .13; *p* < .001). Respondents reported more depressive symptoms (*B* = .05; *SE* = .02; *p <* .05) and presented higher rates of lifetime IPV victimization. Other individual risk and protective factors such as age, gender, sexual orientation, attitudes variables, and substance use variables were not associated with the likelihood of IPV victimization.

**Table 3:** Individual and environmental risk and protective factors of lifetime IPV victimization (N = 600).

Variables			IPV Victimization					
	Model 1				Model 2			
	*B (SE)*	*OR*	*95% CI*	*R* ^*2*^	*B (SE)*	*OR*	*95% CI*	*R* ^*2*^
				.13				.18
Intercept	-2.14 (1.14)	.12	[.01, 1.09]		-2.15 (1.24)	.13	[.01, 1.51]	
Age	.04 (.05)	1.04	[.95, 1.15]		.05 (.05)	1.05	[.95, 1.17]	
Gender (ref = male)	.10 (.23)	1.10	[.71, 1.72]		.07 (.23)	1.05	[.67, 1.66]	
Sexual orientation (ref = heterosexual)	.00 (.00)	1.00	[1.00, 1.00]		.00 (.00)	1.00	[1.00, 1.00]	
Education level	.16 (.15)	1.18	[.87, 1.59]		.18 (.16)	1.20	[.88, 1.64]	
Average monthly income in the past 12 months	.00 (.00)	1.00	[1.00, 1.00]		.00 (.00)	1.00	[1.00, 1.00]	
The number of intimate partners in lifetime	.04 (.03)	1.04	[.98, 1.10]		.03 (.03)	1.03	[.97, 1.09]	
Attitude towards gender roles	-.05 (.03)	.95	[.90, 1.10]		-.04 (.03)	.96	[.91, 1.02]	
Attitude towards IPV	-.02 (.02)	.98	[.94, 1.01]		.00 (.02)	1.00	[.96, 1.04]	
Alcohol consumption	.17 (.09)	1.19	[1.00, 1.41]		.17 (.09)	1.19	[1.00, 1.42]	
Smoking	.09 (.07)	1.09	[.95, 1.25]		.07 (.07)	1.07	[.93, 1.23]	
Depressive symptoms	.05 (.02)*	1.05	[1.01, 1.09]		.03 (.02)	1.03	[.98, 1.07]	
Physical health	-.05 (.03)	.95	[.90, 1.00]		-.04 (.03)	.94	[.88, 1.01]	
Family support					-.01 (.03)	.99	[.93, 1.06]	
Childhood adversities								
Family neglect					-.04 (.02)	.96	[.93, 1.00]	
Witnessed IPV towards male caregiver					.04 (.06)	1.04	[.94, 1.17]	
Witnessed IPV towards female caregiver					.05 (.08)	1.06	[.89, 1.25]	
Experienced physical abuse					.04 (.04)	1.04	[.96, 1.12]	
Experienced sexual abuse					.00 (.00)	1.00	[1.00, 1.00]	
IPV trainings					-.34 (.26)	.71	[.42, 1.19]	
Traumatic events in the past 12 months					.36 (.26)	1.44	[.85, 2.40]	

Note. R^2^ values for multiple logistic regression were calculated as Nagelkerke’s pseudo R^2^ values.*: *p* < .05.

Model 2 added environmental risk and protective variables such as adverse childhood experiences, IPV training, and recent traumatic events. Results indicated these variables accounted for 17.7% of the variance of lifetime IPV victimization (pseudo *R*^*2*^ = .18; *p* < .001). Model 2 was statistically significant and the *R*^*2*^ increased 4.4% of the variance (Δpseudo*R*^*2*^ = .044) over that of Model 1. However, none of the individual and environmental risk and protective factors in model 2 were associated with lifetime IPV victimization.

### Individual and environmental risk and protective factors of IPV perpetration

Table 4 displays the results of the two-step hierarchical logistic regression analysis that explored individual and environmental risk and protective factors of lifetime IPV perpetration. Model 1 included identical individual variables to the model analyzing predictors of lifetime IPV victimization above: sociodemographic variables, attitude variables regarding gender roles and IPV, substance use variables, and health variables. The model with individual characteristics accounted for 21% of the variance in lifetime IPV perpetration (pseudo*R*^*2*^ = .21; *p* < .001). Respondents who presented traditional norms on gender roles (*B* = -.09; *SE* = .03; *p <* .01), who held higher myths on IPV (*B* = -.05; *SE* = .02; *p <* .05), who consumed more alcohol (*B* = .21; *SE* = .09; *<* .05) and who reported poorer physical health (*B* = -.09; *SE* = .03; *p <* .01) were more likely to have experienced IPV perpetration. On the other hand, age, gender, sexual orientation, education level, income, the number of intimate partners, tobacco use, and depressive symptoms were not associated with the likelihood of IPV perpetration.

**Table 4:** Individual and environmental risk and protective factors of lifetime IPV perpetration (N = 600).

Variables			IPV Perpetration					
	Model 1				Model 2			
	*B (SE)*	*OR*	*95% CI*	*R* ^*2*^	*B (SE)*	*OR*	*95% CI*	*R* ^*2*^
				.21				.23
Intercept	-2.41 (1.23)	.11	[.01, 1.27]		-2.01 (1.32)	.17	[.01, 2.25]	
Age	.10 (.05)	1.11	[1.00, 1.23]		.10 (.06)	1.11	[1.00, 1.24]	
Gender (ref = male)	.35 (.24)	1.39	[.87, 2.26]		.32 (.25)	1.36	[.84, 2.21]	
Sexual orientation (ref = heterosexual)	.00 (.00)	1.00	[1.00, 1.00]		.00 (.00)	1.00	[1.00, 1.00]	
Education level	-.07 (.16)	.94	[.68, 1.28]		-.07 (.16)	.94	[.68, 1.29]	
Average monthly income in the past 12 months	.00 (.00)	1.00	[1.00, 1.00]		.00 (.00)	1.00	[1.00, 1.00]	
The number of intimate partners in lifetime	.05 (.03)	1.05	[.99, 1.11]		.05 (.03)	1.05	[.99, 1.11]	
Attitude towards gender roles	-.09 (.03)**	.91	[.86, .97]		-.08 (.03)*	.93	[.87, .98]	
Attitude towards IPV	-.05 (.02)*	.95	[.91, .98]		-.03 (.02)	.97	[.93, 1.01]	
Alcohol consumption	.21 (.09)*	1.23	[1.02, 1.48]		.21 (.10)*	1.23	[1.02, 1.49]	
Smoking	.03 (.07)	1.02	[.88, 1.18]		.02 (.08)	1.01	[.87, 1.17]	
Depressive symptoms	.04 (.02)	1.05	[1.00, 1.09]		.03 (.02)	1.03	[.99, 1.08]	
Physical health	-.09 (.03)**	.89	[.83, .95]		-.09 (.03)**	.90	[.83, .96]	
Family support					.01 (.03)	1.01	[.95, 1.08]	
Childhood adversities								
Family neglect					-.04 (.02)*	.96	[.92, 1.00]	
Witnessed IPV towards male caregiver					-.02 (.06)	.98	[.87, 1.10]	
Witnessed IPV towards female caregiver					.12 (.09)	1.13	[.95, 1.35]	
Experienced physical abuse					-.04 (.04)	.96	[.88, 1.03]	
Experienced sexual abuse					.00 (.00)	1.00	[1.00, 1.00]	
IPV trainings					-.36 (.28)	.69	[.40, 1.20]	
Traumatic events in the past 12 months					.14 (.28)	1.15	[.66, 1.98]	

Note. R^2^ values for multiple logistic regression were calculated as Nagelkerke’s pseudo R^2^ values

*: *p* < .05

**: *p* < .01.

Model 2 added environmental risk and protective variables such as adverse childhood experiences, IPV training, and recent traumatic events. It accounted for 23% of the variance of lifetime IPV perpetration (pseudo *R*^*2*^ = .23; *p* < .001). Model 2 was statistically significant and the *R*^*2*^ increased 2.1% of the variance (Δpseudo*R*^*2*^ = .02) compared to Model 1. In Model 2, among the individual characteristics, traditional views about gender roles (*B* = -.08; *SE* = .03; *p <* .05), alcohol use (*B* = .21; *SE* = .10; *p <* .05), and poorer physical health (*B* = -.09; *SE* = .03; *p <* .01) were positively associated were associated with IPV perpetration. Among environmental factors, family neglect (*B* = -.04; *SE* = .02; *p <* .05) was negatively associated with IPV perpetration.

## Discussion

Based on a nationally representative South Korean young adult sample by their age, gender, and region, this study found that about three in 10 Korean young adults had experienced at least one incidence of IPV victimization and perpetration in their lifetime. About one in four of them had experienced both. The incidence rates supported the previous understandings of the magnitude of IPV and that Korean young adults face heightened risks of IPV in their 20 [1,2]. Lifetime IPV rates in this study were higher than rates of IPV victimization (physical abuse: 12% and emotional abuse: 17.3%) and perpetration (physical abuse: 4.1% and emotional abuse 21.2%) that were reported by Korean men aged 19 to 89 in the 2010 National Survey of Domestic Violence [1]. Co-experience of IPV victimization and perpetration among young adults in this study was also consistent with high chance of mutual violence or bidirectional violence among young adults across the culture [9,35–37]. To address the risk of co-experiencing IPV victimization and perpetration, IPV programs tailored to address both unidirectional and bidirectional IPV for young adult men and women are essential.

In this study, environmental risk factors that were measured by various types of ACEs minimally explain IPV victimization and perpetration among Korean young adults by disconfirming previous knowledge of strong connections between ACEs and IPV. For examples, in young adult or college student samples, intergenerational transmission of IPV by witnessing parental IPV or experiencing abuse by a caregiver [29] and revictimization of IPV among trauma survivors with sexual violence victimization experience in their childhood [38] have been found and supported across cross cultural contexts. Moreover, among Korean young adults, witnessed IPV between caregivers and direct experience with maltreatment were the most important risk factors of both IPV victimization and perpetration [11,13,14]. One speculation about the minimal connections between ACEs and IPV in this study is that it was related to the role of attitudes towards IPV or other belief systems in mediating the associations between ACEs and IPV. A scoping review of 32 studies reviewed the relationships between ACEs and IPV in adulthood including a mediating role of attitudes towards IPV or belief systems [39]. In two reviewed studies, attitudes towards IPV and belief systems in relationships (e.g., fear of abandonment and internalization of emotional deprivation) mediated the relationships between ACEs (e.g., witnessing and experiencing parental and community level violence) and IPV victimization and perpetration among emerging adult samples [40,41]. Yet, these mediating roles were only confirmed regarding cyber abuse in the U.S. context. Existing studies based on Korean young adult samples are outdated and did not examine such a mediating role specific to IPV. This is an explorative study; future studies are needed to explore the mediating mechanisms of individual attitudes and beliefs in relationships on the connections between ACEs and IPV.

Family neglect was the only variable that explained IPV perpetration. Yet, the finding contradicted the hypothesis: a greater experience with family neglect in childhood decreased the likelihood of IPV perpetration in this sample. This finding also contradicted prior evidence that indicated a positive relationship between family neglect experience and IPV perpetration [17] or statistically insignificant relationships between them [42]. Growing up in a family environment where physical and emotional neglect occurred or were severe influenced lack of self-esteem young adults [43], and low self-esteem has been closely associated with elevated risks of IPV victimization [44,45] and IPV perpetration [46,47] in research with broader age ranges. The negative association between family neglect and IPV perpetration in Korean young adults in this study needs further assessment.

As a protective environmental factor of IPV, family support in childhood had no link to IPV in Korean young adults in this study. However, a lack of previous studies about IPV in adulthood’s connection to family support makes it more complicated to meaningfully discuss the finding. A few studies that examined both risk and protective factor of IPV in adulthood have investigated community-level support [48,49] or childhood parental factors such as educational level and income [50]. It has been shown, however, that a nurturing and supportive family environment via warm parenting and child development knowledge mitigates the impact of violence exposure on children and youth [51,52]. Future research should further investigate the effect of family support on IPV, especially given IPV is a critical adverse experience among young adults.

Apart from family neglect, there were individual factors linked to higher chances of IPV victimization or perpetration. For example, greater depressive symptoms were significantly associated with IPV victimization. Depressive is a primary mental health issue that young adult IPV survivors’ report [53]. In previous studies, mental health was associated with IPV victimization for both young adult men and women, and this association was much more illuminated than the association between physical health and IPV perpetration [54–56]. As only one prior study explored the association between depressive symptoms and IPV among Korean young adults, and the only statistically significant result was on IPV perpetration, and only among college students [17], this study’s findings of the relationship between mental health and IPV victimization added significance to our understanding of the need to support Korean young adults’ mental health.

Regarding risk factors of IPV perpetration, attitude variables, both traditional mindset with respect to gender roles and a lack of awareness of IPV predicted IPV perpetration, as did poor physical health. These findings highlighted the crucial roles of personal values and norms in determining IPV among young adults. Previous studies with Korean college student samples revealed that less belief in a rigid gender binary and more egalitarian attitudes towards gender roles decreased the risk of IPV victimization and perpetration [19,57]. This study’s findings with respect to non-permissive attitudes towards IPV aligned with research on single Korean men aged 19–59 years, which showed that their attitude towards IPV was a more crucial determinant of IPV perpetration than personality variables such as self-esteem and borderline personality [58]. Consistent with these previous findings, this study’s findings underscored that helping Korean young adults reconsider their assumptions about gender roles and IPV might be an effective way to prevent IPV perpetration.

This study also found a link between alcohol consumption and IPV perpetration among Korean young adults. These findings aligned with past research showing that alcohol consumption increased IPV perpetration among Korean adult men and women [13] and frequent (more than once a week) and problematic drinking of alcohol was associated with IPV perpetration among Korean college students [59]. Alcohol consumption seems to explain IPV perpetration more than IPV victimization as it explained IPV perpetration for both genders among Korean men and women, but it only explained victimization among Korean men [13] and it did not predict IPV victimization in U.S. college women [60]. Yet, alcohol use predicted sexual abuse victimization among college women [60]. Alcohol drinking, including heavy alcohol drinking, is socially accepted in Korean culture, and until the legal systems provides leniency for crimes conducted under feeble minded conditions, a term often meaning under the influence of alcohol. Other kinds of intoxicants are unusual in Korea. Until 2018, this leniency was unconditional, and a judge could still grant it under the latest law [61]. This study’s findings, along with those of prior research, suggest that this policy may be harmful and perpetuate IPV; IPV prevention approaches should address perpetrator accountability concerning the influence of alcohol and foster dialogue about ensuring safety in intimate relationships by managing problematic alcohol drinking.

Indeed, the findings of this study have practical, research, and policy implications for efforts to prevent IPV among Korean young adults, one of the groups most vulnerable to both victimization and perpetration. For health care practitioners, recognizing and supporting young adult IPV survivors’ needs and addressing their mental health challenges will be beneficial. Development and implementation of IPV prevention programs that address individual risk factors such as alcohol consumption and attitudes towards IPV and about gender roles in institutions of higher education, workplaces, and the military, all of which bring Korean young adults together, have the potential to prevent IPV perpetration. Yet further research is needed to investigate determinants of attitudes towards IPV and toward gender roles to identify interventions to foster non-permissive attitudes about IPV and egalitarian attitudes about gender as part of IPV prevention. Future research also needs to investigate how these adults’ attitudes towards IPV and towards gender roles potentially mediate the relationships between ACEs and IPV. In addition, evidence-based IPV prevention programs that intervene in beliefs or attitudes towards IPV and gender roles among Korean young adults are vital. Replication studies examining the role of environmental risk factors centering on adverse childhood experiences are also needed to support this study’s findings. To address identified risk and protective factors and make IPV prevention feasible, funding support from national to local government offices is vital to address individual risk factors of IPV victimization and perpetration via various levels of prevention efforts (e.g., primary, secondary, tertiary prevention). Legislative reassessment about leniency toward. People who have committed criminal acts “under the influence of alcohol” will be helpful to ensure IPV perpetrator accountability.

### Limitations and strengths

The sampling of this study was well planned to represent the target population. The study used a random sampling strategy considering quota of age, binary gender, and region based on the quota of these characteristics in the population frame. Dominance of female samples has been a chronic issue in IPV research [62], and this study obtained male respondents that are representative of the population. Yet, the study’s findings may be still biased as the study participants voluntarily registered as subjects of the online survey, and their response quality might be less to control compared to conducting survey by a trained interviewer. Response rates of the participants were good or excellent as 49.1% of the participants who opened the invitation email completed the survey. The current study initially invited 3,000 people (five times the target sample size). Considering the high response rate and quick responses (target sample size was reached in a few days), inviting a smaller number (e.g., three times the target sample size instead of five times) would have helped us include dropped responses after reaching the target sample size. This study defined young adults as being aged 19–26 [63]. Traditionally, these emerging adults were expected to “become financially independent, to establish romantic relationships, and become parents” [64], but diversity in relationship status in the study’s sample was very low due to changing social norms about these transitions, delays in marriage, and emphasis on education during this developmental stage. This study may not illuminate the experiences of married Korean young adults because they were underrepresented in the sample given the sample’s age.

## Conclusion

This study revealed that about one in three Korean young adults ages 19–26 had ever experienced IPV victimization or IPV perpetration within, on average, 3.6 intimate relationships. Using person-in-environment and life course perspective of cumulative adversities, the study identified depressive symptoms as a factor explaining lifetime IPV victimization and other individual factors such as attitudes about gender roles and IPV, alcohol consumption, and physical health that predicted IPV perpetration. No associations between most of the environmental risk factors such as witnessing parental IPV or abuse experience and IPV were contradictory to previous findings (e.g., [11]) that underscore intergenerational transmission of IPV and long-term adverse health effect of IPV among young adults. This study’s findings highlight the importance of addressing Korean young adults’ IPV via diverse prevention program approaches including gender inclusive programs, mental health supporting programs for IPV survivors, and culturally tailored IPV prevention programs and evaluation research that promote equality in gender role and awareness of IPV and knowledge of the potential impact of alcohol use in intimate relationships.
